# The Mediating Role of Romantic Attachment in the Relationship Between Attachment to Parents and Aggression

**DOI:** 10.3389/fpsyg.2019.01824

**Published:** 2019-08-06

**Authors:** Alessandra Santona, Paola De Cesare, Giacomo Tognasso, Massimo De Franceschi, Andrea Sciandra

**Affiliations:** ^1^Department of Psychology, University of Milano-Bicocca, Milan, Italy; ^2^Department of Psychology, Sapienza University of Rome, Rome, Italy; ^3^Family Counseling Service “Casa di Varese”, Varese, Italy; ^4^StarLab, Socio Territorial Analysis and Research, University of Padua, Padua, Italy

**Keywords:** attachment, aggression, parents, romantic attachment, attachment to parents

## Abstract

**Background:**

A secure attachment style could promote more intimacy in romantic relationships, while an insecure attachment style could be correlated with less positive romantic relationships in adulthood. Numerous studies have noted that a secure attachment to parents was correlated with lower levels of aggression, whereas insecure attachments were associated with higher levels of aggression. We aimed to investigate the role of the attachment system as a mediator of the expression of aggressiveness during adolescence. Specifically, we considered that the attachment to parents and peers could influence one’s attachment to a romantic partner.

**Methods:**

We empirically tested whether there were relationships of parent and peer attachment on aggressiveness mediated by romantic attachment style. Participants of the study included 411 students.

**Results:**

Results indicated that for males an insecure father-child attachment style seems to be associated with higher levels of anxiety and avoidance in romantic attachments and then with aggressiveness. For females, an insecure mother-child attachment style seems to be associated with higher levels of aggressiveness.

**Conclusion:**

The attachment to parents and to peers plays a key role in defining romantic attachment according to gender, and these dimensions in turn tend to affect the levels of aggressiveness.

## Introduction

Adolescence is a specific stage of the life cycle characterized not only by physical and biological changes but also by significant psychological, cognitive and social transitions (Sarracino et al., 2011; Gray et al., 2012). From the *physical* development point of view, teenagers enter the stage of puberty, which is characterized by internal (both hormonal and neural; Spear, 2000; Adams and Berzonsky, 2003) and external changes (physical development). *Cognitive* development increases adolescents’ ability to process information, and it improves their decision-making, self-control and emotional regulation skills. These changes are accompanied by developments in defining one’s own sense of identity, which includes the ability to understand oneself, self-esteem and self-concept (Wong et al., 2010; Tanti et al., 2011; Gray et al., 2012; Syed and Seiffge-Krenke, 2013). Emotional regulation processes are crucial for adolescents’ development (Wong et al., 2010; Tanti et al., 2011; Syed and Seiffge-Krenke, 2013; Santona and Tognasso, 2017).

Since attachment theory was formulated by Bowlby (1969) and the process through which children create emotional bonds with their caregivers was described, many attempts have been made to discover the effect of the quality of parent–infant relationships on the future development of children (DeKlyen and Greenberg, 2008; Riem et al., 2017). Many researchers underline the key role that parents play in the emotional regulation of their sons (Southam-Gerow and Kendall, 2002; Sheffield Morris et al., 2007; Calkins and Leerkes, 2011). Studies (Mikulincer and Shaver, 2009) in this field have focused on the establishment of secure bonds with the main caregivers and underlined that these bonds promote the development of characteristics such as self-esteem or emotion regulation. In contrast, internal working models (MOI) that are developed in an insecure relationship are characterized by distrust, anger and insecurity in relationships with other people (Sheffield Morris et al., 2007; Gallarin and Alonso-Arbiol, 2012). Ultimately, the parents’ function is to sustain their sons’ abilities to cope with the emotions that they experience in their lives. According to many researchers (Cassidy, 1994; Shaver and Mikulincer, 2002), in practice, people with a secure attachment style may have better emotion regulation than people with an insecure/avoidant attachment style, who tend to deactivate or to suppress their emotions, and people with an insecure/anxious attachment style, who tend to present heightened emotional reactions.

Due to the physical and cognitive changes that affect their social life, adolescents begin to transfer the functions of attachment to peers: adolescents will rely mainly on their peer groups, trying to distance themselves from their early attachment figures (Venta et al., 2014). They focus their energies on managing their friendships and on their first romantic relationships rather than on conserving their parental relationship (Allen et al., 2007). Usually, the emergence of new autonomy within an attachment relationship with caregivers allows the adolescent to focus on new significant relationships, such as peer groups and especially romantic relationships. The main goal of an adult romantic relationship is to integrate both the care and protection dimensions and to achieve a symmetrical relationship with the romantic partner. Each partner is identified as a secure base and as a main attachment figure for the other, and both partners alternate in the role of who takes care *of* and who receives care *from* the other partner (Carli et al., 2009; Pace et al., 2015).

## Attachment to Parents, Friendships and Romantic Relationships

Studies in this field that focus on adolescence (Ammanniti et al., 2000; Furman et al., 2002; Sroufe, 2005; Allen et al., 2007; Auslander et al., 2009; Doyle et al., 2009; Furman and Collins, 2009) have highlighted *the relationship between one’s style/model of attachment to parents and the quality of friendships and romantic relationships in adolescence*. According to many studies (Collins et al., 2002; Nickerson and Nagle, 2005; Nosko et al., 2011; Tarabulsy et al., 2012; Karakurt et al., 2013), a secure attachment style could promote more intimacy in romantic relationships, while an insecure attachment style could be correlated with less positive romantic relationships in adulthood. Pascuzzo et al. (2013) underlined that only people with an anxious attachment style perpetuated the same bond within their romantic relationships. In contrast, Holman et al. (2009) affirmed that some people with an insecure attachment to their parents – especially those with avoidant attachment – worried about their partner’s availability. Similarly, Doyle et al. (2009) stated that an insecure attachment to one’s mother or best friend was associated with an insecure attachment to one’s partner, while an insecure attachment with one’s father was associated with an insecure attachment with friends. These assertions have been supported by several studies (Crowell et al., 1999; Furman et al., 2002; Mayseless and Scharf, 2007) that compared retrospective assessments of early attachment bonds to more specific assessments of actual attachment bonds with a partner.

## Attachment to Parents and Peers and Aggression

Aggression refers to attacking another in a hurtful way (Buss, 1966), and this includes both physical and psychological harm (Bandura, 1973). The expression of aggression may be affected by adolescents’ abilities to regulate their emotions. As mentioned, the regulation of emotions originates not only from biological and psychological factors but also from the influence of parents and other social figures on the adolescent during his or her growth (Pascuzzo et al., 2013). Parents, in fact, help their sons identify, endure and manage their emotions through their validation of these emotions (Sheffield Morris et al., 2007). Regulating emotions implies the ability to react in a flexible and socially appropriate manner to stressful events or negative emotional experiences. It is important to emphasize that high levels of emotional responsiveness – caused by an inability to regulate emotions – can predict aggressive behaviors during childhood. High levels of emotional responsiveness can also predict aggressive, socially deviant and anti-social behaviors during adolescence and adulthood (Gallarin and Alonso-Arbiol, 2012). Several studies (Jimenez et al., 2005; Sheffield Morris et al., 2007; Stocker et al., 2007; Farrington, 2009) have shown that the quality of the adolescent-family relationship is linked to the expression of aggression. Specifically, *insecure attachment to one’s parents has a significant role in eliciting aggression in adolescents* (Karakurt et al., 2013). Numerous studies have noted that a secure attachment to parents was correlated with lower levels of aggression (Laible et al., 2000; Karakurt et al., 2013), whereas insecure attachments were associated with higher levels of aggression (Brown and Bakken, 2011). The secure attachment style, then, is an essential moderator in the expression of aggression during adolescence (Taubner et al., 2013).

However, the influences of the mother and father separately have not been investigated in many studies (Doyle and Markiewicz, 2005; Bosmans et al., 2006; Murray et al., 2014). Gallarin and Alonso-Arbiol (2012) found that only the attachment to the father influenced adolescent’s expressions of aggression: an insecure attachment to the father predicted higher levels of aggression during adolescence. These results confirmed previous studies (Kuterovac-Jagodic and Kerestes, 1997; Liu, 2008) in which an insecure attachment to the father was one of the primary causes of the future development of externalizing symptoms (such as aggression). Furthermore, Wright (2015) found that there was a significant link between the quality of the mother-adolescent attachment and the expression of aggression in the adolescent’s romantic relationships. In fact, adolescents with an insecure attachment to their mother tended to develop anxious attachments to their partner and to show aggressive behaviors in their relationships. Wright’s (2015) study confirmed the findings of many studies (Simpson et al., 1996; Cummings-Robeau et al., 2009; Miga et al., 2010; Wright, 2015) that both an anxious and an avoidant attachment style were risk factors for the expression of aggression toward one’s partner.

Many studies (Burton et al., 2013; Karakurt et al., 2013; Wright et al., 2015) have found that peer groups also play an essential role in eliciting aggression in adolescents. Thus, it seems that the most important risk factor for the expression of anti-social behaviors in adolescence is the influence that a peer group exerts on its members (Zara and Farrington, 2009; Casey and Beadnell, 2010). Moreover, Brown and Bakken (2011) stated that attachment to peers seemed to influence antisocial behavior. Laible et al. (2000) also demonstrated that attachment to peers affected aggression; additionally, the authors showed that this influence appeared to be greater than that of attachment to parents. Similarly, Wright et al. (2015) reported that adolescents with higher scores on the assessment of attachment to peers were less involved in aggressive behaviors. Conversely, lower scores on the attachment to peers assessment were a significant risk factor for the emergence of threatening or aggressive behaviors. Burton et al. (2013) confirmed these results, highlighting the fact that a secure attachment style is an essential protective factor against involvement in bullying acts.

Previous research (Watt et al., 2016; You and Kim, 2016; McDermott et al., 2017; Nakamura and Kawabata, 2018) focused their attention on the difference between male and females in the relationship between attachment and aggression. Specifically, Nakamura and Kawabata (2018) underlined that anxiously attached males displayed higher levels of both proactive and reactive aggression than anxiously attached females, who showed only proactive aggression. Moreover, You and Kim (2016), conducted a study of a sample of adolescents and found that females with higher levels of parent and peer attachment, higher levels of empathy and higher levels of self-control presented lower levels of aggression than males, whereas only peer attachment was negatively associated with aggression for males. Finally, Watt et al. (2016) suggested that men report more attachment avoidance than women and higher levels of aggression.

## Aims of This Study

**FIGURE 1:**
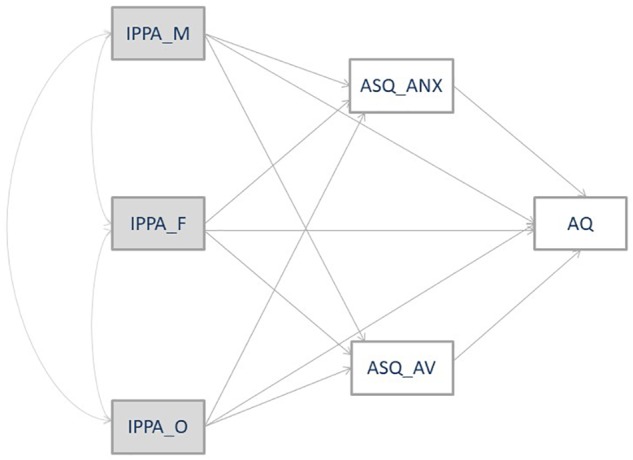
Hypothetical model. Dependent variables: Aggression (AQ), Anxiety Attachment Style (ASQ_ANX), and Avoidance Attachment Style (ASQ_ANX). Independent variables: Maternal Attachment (IPPA_M), Paternal Attachment (IPPA_F), and Close Friend Attachment (IPPA_O).

Based on the publications and research presented above, we aimed to investigate the relationship between attachment and aggression:

(1) First, we investigated whether attachment to parents and peers could influence one’s attachment to a romantic partner (*Hypothesis 1*).
(2) Second, we focused our attention not only on infant attachment – assessing both maternal and paternal attachment – but also on the mediating role that romantic attachment could have in the expression of aggressive behaviors (*Hypothesis 2*).
(3) Third, we empirically tested whether there were direct and indirect relationships of parent and peer attachment with aggression mediated by romantic attachment (*Hypothesis 3*) (Figure 1).

To address this issue, separate path analysis models for both boys and girls were used to test (a) whether attachment negatively influenced aggression, (b) whether attachment negatively influenced romantic attachment style, and (c) whether romantic attachment style had a positive correlation on aggression. More specifically, we began with a fully saturated model, including all the variables and the described paths.

## Materials and Methods

### Participants

The participants were 411 students attending different schools in two major Italian cities, Rome (*N* = 193) and Milan (*N* = 218); 243 were female, and 168 were male. The ages of the students were between 14 and 18 years, with an average age of 16.85 years (*SD* = 1.41). Specifically, the females had an average age of 17 years (*SD* = 1.46), while the males had an average age of 16.6 years (*SD* = 1.28). The analyses showed a significant difference between the average age of the females and that of the males [*T*(409) = -3.007, *p* = 0.002; α = 0.05].

Additionally, 62.5% of the participants said they did not have an ongoing romantic relationship, while 37.5% said they were in a meaningful relationship that had lasted an average of 13.51 months.

### Instruments

#### Perceptions of the Emotional and Cognitive Dimensions of Family and Peer Relationships

The *Inventory of Parent and Peer Attachment* (IPPA) (Greenberg et al., 1983; Armsden and Greenberg, 1987, 1989) is a self-report questionnaire that was constructed for people between 12 and 19 years old to assess and estimate their perceptions of the emotional and cognitive dimensions of family and peer relationships. It explores adolescents’ attitudes toward their attachments bonds with their most important affective people: their mother, father and peers. These individuals were selected because adolescents perceive them as sources of psychological security. The IPPA has 3 different scales (25 items each). The adolescents rate their agreement with each item on a 5-point Likert-type scale (*1* = *never or almost never true, 2* = *not very often true, 3* = *sometimes true, 4* = *often true, 5* = *always or almost always true*). In addition to an overall attachment security score, the instrument provides a separate score for all three affective people on each of the following subscales: Trust, Communication and Disaffection. According to the framework of attachment theory, the score on the disaffection scale is reversed, and thus a low score corresponds to a high level of attachment security and vice versa.

The validation of the Italian version of the IPPA was performed by San Martini et al. (2009).

In our sample, the internal consistency of the maternal version was α = 0.819; for the paternal version, it was α = 0.819, and for the peer version, it was α = 0.864.

#### Romantic and Adult Attachment

The *Attachment Style Questionnaire* (ASQ) (Feeney et al., 1994b) is a self-report questionnaire that aims to measure individual differences in adult attachment through two dimensions: a*nxiety* and *avoidance*. It enables individuals to be classified according to their level of anxiety and avoidance in four categories: *secure, avoidant, preoccupied*, and *fearful*. The ASQ consists of 40 items that are rated on a 6-point Likert-type scale (*1* = *Completely disagree; 2* = *Mostly disagree; 3* = *Slightly disagree; 4* = *Slightly agree; 5* = *Mostly agree; 6* = *Completely agree*). Based on theoretical expectations and principal component analysis, 5 scales were identified: *Confidence* (consisting of 8 items; this scale assesses the respondent’s confidence in themselves and others and reflects a secure attachment style); D*iscomfort with Closeness* (consisting of 10 items; it represents the core of the attachment avoidance style); *Need for Approval* (consisting of 7 items; it indicates the degree of need for acceptance and confirmation from other people and corresponds to worried and fearful styles); *Preoccupation with relationships* (consisting of 8 items; it includes the tendency toward anxious and dependent relationships that characterize anxious/ambivalent attachment); and *Relationships as Secondary* (consisting of 7 items; it indicates a gap in intimate relationships). As indicated by Feeney et al. (1994a), the ASQ is a valid instrument for assessing adult and romantic attachment during adolescence. In Italy, the ASQ has been validated by Fossati et al. (2003a). In our sample, the internal consistency was α = 0.768.

#### Aggression

The *Aggression Questionnaire* (AQ) (Buss and Perry, 1992) is a self-report questionnaire developed to assess aggression. The final structure of the AQ includes 29 items rated on a Likert-type scale ranging from 1 to 5 (*1* = *never or almost never true, 2* = *not very often true, 3* = *sometimes true, 4* = *often true, 5* = *always or almost always true*). The AQ consists of 4 factors: Physical aggression, Verbal aggression, Anger, and Hostility. Adding the scores of all items provides a Total Aggression score that can range between 29 and 145. The Italian version of the AQ was developed by Fossati et al. (2003b). In our sample, the internal consistency was α = 0.823.

### Procedure

We administered a battery of eight tests to each student during the school day in addition to a list of questions specifically prepared to obtain general information about their family composition. Before the students began completing the questionnaires, a research assistant briefly explained the content and meaning of the study, and he remained available for any questions during the 40 min needed to complete the questionnaire. All the questionnaires were anonymous, and the students were informed that they could stop participating in the study tasks at any time. The order of the administration of questionnaires was varied to avoid the risk of a systematic effect on the responses. The questionnaires were administered in two sessions after the authorization to use personal data was received in accordance with the Italian Privacy Law n. 675/96. Before the students began completing the questionnaires, written informed consent was obtained from both parents of each participant.

The subjects were asked to complete the questionnaires in the most thorough and individualized manner possible. All the students’ questionnaires were digitally codified and scored based on the instructions provided by the author of each scale.

This research did not receive any specific grant from funding agencies in the public, commercial, or not-for-profit sectors.

The research project was previously approved by the Ethics Committee of the psychology department of Milano-Bicocca University.

### Statistical Analyses

First, we conducted preliminary analyses of variance (ANOVAs) on the mother, father and close friend measurements of attachment (IPPA), the anxiety and avoidance dimensions of ASQ and AQ according to the adolescent’s gender. Following Cohen’s suggestions (Cohen, 1992), partial eta-square estimates were only considered to be substantially significant with 1–5% effect sizes and a significance level of 5%. We also tested the multivariate gender means difference using a MANOVA (with Pillai-Bartlett trace) that included the IPPA, ASQ, and AQ.

Second, the relationships among variables were explored via Pearson’s correlations that were computed separately for girls and boys.

Third, we estimated path analytic models with LISREL for both boys and girls to test whether the relationships between attachment (IPPA) and AQ were mediated by ASQ. LISREL 8 (Jöreskog and Sörbom, 1996) was used to assess the model fit. Multiple criteria must be considered when evaluating model fit, and it is determined on the basis of various simultaneous measures, the first of which is chi-square (χ^2^). A model fits the data well when χ^2^ is not significant (*p* ≥ 0.05). This statistic, however, is sensitive to sample size; this can lead to the rejection of a model that differs very slightly from the data for large samples, and conversely, it can result in the acceptance of a model with salient differences in the data for small samples. Therefore, we followed Schermelleh-Engel et al.’s (2003) suggestions, which consider a chi-Square/d.f. ratio lower than 3 to be adequate. The fit of the model was also assessed with the comparative fit index (CFI), the non-normed fit Index (NNFI), and the root mean square error of approximation (RMSEA) (Kline, 2005). A CFI of 0.95 or above indicates a good fit, and a value below 0.90 indicates a poor fit. Additionally, NNFI values greater than or equal to 0.95 indicate a good fit. If the RMSEA index is less than or equal to 0.05, the model is considered a good fit; values between 0.05 and 0.08 suggest a reasonable error of approximation, and if the index is greater than or equal to 0.10, the model is considered a poor fit.

The significance of the standardized path coefficients was determined by comparing the (absolute) t ratio to a critical t of 1.96 (*p* ≤ 0.05).

Therefore, the overall fit of the models was determined by a combination of results from the fit indexes, the significance of the standardized path coefficients, and the significance of the indirect effect.

To determine the influence of the grouping variable (gender), we followed the suggestions of Jöreskog and Sörbom (1996); that is, the path analysis model was fitted separately to the two datasets. A model that was identical across genders would indicate that we should assess whether the factor loadings of the model were invariant across the two groups. Otherwise, we should theoretically analyze the differences between the two models.

## Results

To assess the gender difference in mean scale values, we used a MANOVA test. The results are reported in Table 1.

**Table 1:** Means and Standard Deviations for mother, father and close friends attachment (IPPA), anxiety and avoidance dimensions of attachment style questionnaire (ASQ) and aggression questionnaire (AQ) according to gender (*N* = 493).

	Gender								
	
	Male (*n* = 189)		Female (*n* = 304)		ANOVA			MANOVA		
				
	*M*	*SD*	*M*	*SD*	*F*(1,492)*^a^*	*P*	$\eta_{p}^{2b}$	*F*(6,486)*^c^*	*P*	*Pillai trace*
IPPA_M	84.07	12.53	86.50	15.55	3.28	0.07	0.01			
IPPA_F	87.44	19.78	76.33	13.87	53.68	0.00	0.10			
IPPA_O	84.10	14.07	96.14	13.36	90.89	0.00	0.16			
ASQ_ANX	-0.73	1.72	-0.72	1.52	0.01	0.95	0.00			
ASQ_AV	-0.49	1.35	-0.83	1.46	6.64	0.01	0.01			
AQ	83.98	14.25	77.81	15.28	45.77	0.00	0.09			
								29.63	0.00	0.27


*^*a*^*F*-value with degrees of freedom. ^*b*^$\eta_{p}^{2b}$ measure of effect size. ^*c*^Approximative *F*-value with degrees of freedom.*

MANOVA showed a significant difference between the male and female adolescents (*p* < 0.001). More precisely, significant gender differences were found in the attachments to the father and romantic partners, the avoidance romantic attachment style (assessed by the ASQ) and aggression. The avoidance romantic attachment style, in particular, showed an effect size equal to 1%.

The correlation matrices for the two genders are reported in Tables 2, 3. We found several significant correlations for both genders, with low and medium effect sizes (Cohen, 1988). Informal comparisons among the two matrices revealed a clear gender-specific correlation structure.

**Table 2:** Correlations of the parent and peer attachment (IPPA), anxiety and avoidance dimensions of attachment style questionnaire (ASQ) and aggression questionnaire (AQ) for male^a^ adolescents.

	IPPA_M	IPPA_F	IPPA_O	ASQ_ANX	ASQ_AV	AQ
IPPA_M	-	-	-	-	-	-
IPPA_F	0.49^***^	-	-	-	-	-
IPPA_O	0.25^***^	0.05	-	-	-	-
ASQ_ANX	-0.00	-0.16^*^	0.13	-	-	-
ASQ_AV	-0.19^**^	-0.16^*^	-0.38^***^	0.11	-	-
AQ	-0.05	-0.08	0.08	0.26^***^	0.20^**^	-


*^∗^*p* < 0.05; ^∗∗^*p* < 0.01; and ^∗∗∗^*p* < 0.001. ^*a*^*n* = 189.*

**Table 3:** Correlations of the parent and peer attachment (IPPA), anxiety and avoidance dimensions of attachment style questionnaire (ASQ) and aggression questionnaire (AQ) for female^b^ adolescents.

	IPPA_M	IPPA_F	IPPA_O	ASQ_ANX	ASQ_AV	AQ
IPPA_M	-	-	-	-	-	-
IPPA_F	0.24^***^	-	-	-	-	-
IPPA_O	0.10	0.06	-	-	-	-
ASQ_ANX	-0.11^*^	-0.06	-0.30^***^	-	-	-
ASQ_AV	-0.03	-0.09	-0.47^***^	-0.30^***^	-	-
AQ	-0.20^**^	-0.05	-0.21^***^	0.37^***^	0.20^**^	-


*^∗^*p* < 0.05; ^∗∗^*p* < 0.01; and ^∗∗∗^*p* < 0.001. ^*b*^*n* = 304.*

Given the gender difference identified by the MANOVA and the very different correlation matrices, two separate path analyses were used to evaluate the relationships between the IPPA, ASQ, and AQ scores and the role of the ASQ as a mediator between the IPPA and AQ. Specifically, we started with a fully saturated model that included all of the variables (see Figure 1). Path coefficients were removed based on their significance.

**FIGURE 2:**
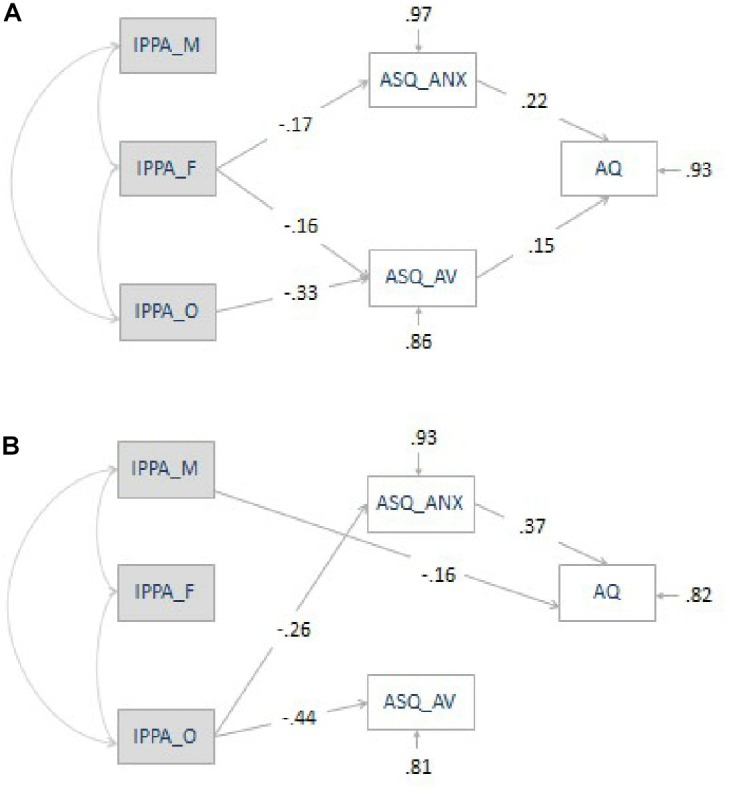
**(A,B)** Final path analytical models of the effects of parent/peer attachment (IPPA) and anxiety/avoidance dimensions of attachment style questionnaire (ASQ) on aggression questionnaire (AQ) in adolescent boys **(A)** and girls **(B)**. Coefficients are standardized structural coefficients. All coefficients are significant at least above the *p* < 0.05 level. Maternal attachment (IPPA_M) did not have any significant causal relationships with any other variable in model **(A)**. Paternal attachment (IPPA_F) did not have any significant causal relationships with any other variable in model **(B)**.

Figure 2A shows the final path analytic model for the male adolescents. In this model, paternal attachment was negatively associated with both the anxiety and avoidance dimensions of romantic attachment style (i.e., a higher level of paternal attachment resulted in lower levels of anxious and avoidant romantic attachment styles, which in turn were positively associated with aggression). Thus, both the anxiety and avoidance dimensions of romantic attachment style acted as mediators between paternal attachment and aggression. Indirect effects were estimated statistically as the product of standardized direct effects (significant path coefficients). For the male adolescents, paternal attachment had an indirect effect on aggression equal to -0.06. In this model, the aggression level was expected to decrease by approximately 0.06 standard deviations for every increase of one full standard deviation in paternal attachment via its prior effect on anxious and avoidant romantic attachment. Moreover, peer attachment had an indirect effect equal to -0.05 on aggression through avoidant romantic attachment style. Maternal attachment did not have any significant relationships with any other variable, so there was no causal path from maternal attachment to any independent variable. Attachment to peers was also negatively associated with the avoidant romantic attachment style, and thus, its relationship to aggression was mediated by the avoidant attachment style. All these paths were significant (*p* < 0.05), and the model fit the data reasonably well as indicated by multiple indicators of fit: chi-square/d.f. ratio = 14.21/10 = 1.421, RMSEA = 0.046, CFI = 0.96, and NNFI = 0.95. The model accounted for 3%, 14% and 7% of the variance in anxious and avoidant attachment styles and aggression, respectively.

Figure 2B shows the final path analytic model for the female adolescents. In this model, maternal attachment had only a negative direct effect on aggression (i.e., a higher level of maternal attachment resulted in a lower level of aggression, while attachment to peers was negatively associated with both the anxious and avoidant dimensions of romantic attachment style, which in turn were positively associated with aggression). Therefore, attachment to peers had an indirect negative effect on aggression that was mediated by the anxious romantic attachment style. All these paths were significant (*p* < 0.001), so we calculated the indirect effect of peer attachment on aggression through the anxious romantic attachment style; the effect was equal to -0.10. In the model for the female adolescents, the aggression level was expected to decrease by approximately 0.10 standard deviations for every increase of one full standard deviation in peer attachment via the prior effect of an anxious romantic attachment style. Paternal attachment did not have any significant relationships with any other variable, so there was no causal path from paternal attachment to any independent variable. This model fit the data reasonably well as demonstrated by multiple indicators of fit: chi-square/d.f. ratio = 16.89/11 = 1.535, RMSEA = 0.043, CFI = 0.96, and NNFI = 0.95. The model accounted for 7, 19, and 17% of the variance in anxious and avoidant attachment styles and aggression, respectively.

## Discussion

The aim of this study was to examine the relationship between attachment and the expression of aggression during adolescence. As recommended by Wright (2015), we focused our attention not only on infant attachment style but also on the mediating role that romantic attachments could have in the expression of aggressive behaviors. Our hypotheses lay the groundwork for the assumption that the child-parent attachment has both a direct and an indirect effect (with romantic attachment as the mediator) on aggression. The results confirmed our expectations: during adolescence, the attachment system seems to play an important role in the expression of aggressive behaviors. In particular, this effect seems to differ by gender.

For male adolescents, an insecure father-child attachment style seems to be associated with higher levels of anxiety and avoidance in romantic attachments and thus with aggression. In the same way, insecure peer attachment seems to be associated with an avoidant style in romantic attachments and thus with higher levels of aggression. For female adolescents, an insecure mother-child attachment style seems to be directly associated with higher levels of aggression. Moreover, an insecure peer attachment style positively affects the anxiety and avoidance dimensions of romantic attachment and indirectly increases the levels of aggression in people with an anxious romantic attachment style.

Overall, attachment to parents and to peers plays a key role in defining the anxiety and avoidance dimensions of romantic attachments according to gender, and these dimensions in turn tend to affect the levels of aggression. Our results are in line with some research in the literature: the father-child attachment bond seems to affect aggressive behaviors (Kuterovac-Jagodic and Kerestes, 1997; Laible et al., 2000; Liu, 2008; Gallarin and Alonso-Arbiol, 2012; Karakurt et al., 2013). Moreover, it seems that the quality of the mother-child attachment could influence the levels of aggression during adolescence, especially in romantic relationships (Wright, 2015), and both anxious and avoidant parent and partner attachment styles were risk factors for the expression of direct aggression toward the partner (Cummings-Robeau et al., 2009).

Our results highlight a complex relationship that has not been accounted for in other studies. More specifically, a more complex framework seems to emerge from our data in which an insecure father-child attachment is crucial for the expression of aggression in male adolescents, whereas an insecure mother-child attachment is crucial for female adolescents. Furthermore, for the first time, we take into account the mediating role that romantic attachment plays in the relationship between attachment and aggression. Regarding the contribution of peer attachment, we need to refer to the stage of the life cycle that adolescents are passing through. During this stage, individuals must gradually separate themselves from parental figures to rely on their peer groups and on their first romantic relationships (Allen et al., 2007; Venta et al., 2014). For this reason, relationships with peers critically affect the expression of aggression in both males and females (Zara and Farrington, 2009; Casey and Beadnell, 2010). Many studies have confirmed that the quality of an adolescent’s relationships with peers is the first risk or protective factor for the expression of antisocial or aggressive behavior (Brown and Bakken, 2011; Burton et al., 2013; Wright et al., 2015).

The findings of our study should be considered in light of attachment theory (Bowlby, 1969). In particular, we need to consider the link between attachment theory and the regulation of emotions. According to attachment theory, an insecure relationship with both parents could lead to worse emotion regulation and therefore a lower ability to control aggression (Sheffield Morris et al., 2007). Many studies highlight the key role played by parents in their sons’ ability to regulate emotions (Sheffield Morris et al., 2007; Calkins and Leerkes, 2011). The quality of the child-parent attachment bond can promote or obstruct the optimal regulation of emotions through more or less suitable emotion regulation strategies. Specifically, both anxious and the avoidant attachment styles seem to be associated with poor emotion self-regulation (Cassidy, 1994; Shaver and Mikulincer, 2002). Our results support the relationship between attachment theory and emotional self-regulation. In fact, this association functions consistently with the literature regarding father-son and mother-daughter relationships, which affirms that during adolescence, boys and girls have different significant parental figures – mothers for females and fathers for males. Moreover, this association confirms that the quality of attachment could affect the expression of aggression. In particular, adolescents with an anxious attachment style could more readily act aggressively than adolescents with a secure attachment style because they tend to *act* when they are in a stressful situation (Wright, 2015). At the same time, aggressive behaviors could be an inappropriate and dysfunctional emotion regulation strategy that is used with the aim of obtaining further contact with the attachment figure (Dutton, 2003). In contrast, adolescents with an avoidant style tend to avoid conflict and to use less overt means of communication. However, according to many studies (Kobak and Sceery, 1988; Mikulincer, 1998), the failure of a “disengagement” strategy could cause anger and aggression.

Our results could be interesting also for clinical interventions in terms of emotion regulation processes. As we said before, many researchers (Cassidy, 1994; Shaver and Mikulincer, 2002) underlined that people with a secure attachment style could have a better emotion regulation than people with an insecure attachment style. In fact, people with an insecure/avoidant attachment style tend to deactivate or to suppress their emotions, whereas people with an insecure/anxious attachment style tend to present heightened emotional reactions. It is important to underline that high levels of emotional responsiveness – caused by an inability to regulate emotions – can predict aggressive behaviors during childhood and aggressive, socially deviant and anti-social behaviors during adolescence and adulthood. In this field, numerous research have highlighted that a secure attachment style was correlated with lower levels of aggression (Laible et al., 2000; Karakurt et al., 2013), whereas insecure attachments (avoidant and anxious) were associated with higher levels of aggression (Brown and Bakken, 2011). The secure attachment style, then, is an essential moderator in the expression of aggression during adolescence (Taubner et al., 2013).

According to this perspective we can use our conclusions to think about prevention programs that could be introduced in schools. The aim of these programs could be to increase the ability to cope with stress, to improve ability to communicate better with friends and parents and increase ability to cope with anger (Dingle et al., 2016; Dingle and Fay, 2017; Waldron et al., 2018). Research (Dingle and Fay, 2017) underlined that this kind of programs could enhance emotion awareness and regulation and could promote higher levels of awareness about feelings and behaviors. Furthermore, these programs could help adolescents to find a better way to express anger and to regulate their emotions.

## Conclusion

In conclusion, we can confirm that attachment to parents seems not only to play a key role in the quality of romantic attachment but to also directly affect the level of aggression during adolescence. In turn, romantic attachment seems to act as a mediator between infantile attachment and the expression of aggression. Because this study was conducted with adolescents, we can highlight the importance of peer group relationships and their role in determining the quality of romantic attachment and thus the expression of aggressive behaviors.

### Limitations and Future Studies

The main limitations of the study should be mentioned as they indicate future directions for research in this field. One possible limitation of the study is our sampling method: because we used a convenience sample, our conclusions cannot be generalized to the entire population. Moreover, because the battery of instruments was administered at the same time for all the participants, our results may be marred by a social desirability bias. Additional research with a larger sample size would enable a stratified analysis by class age as well as a deeper investigation of the differences in the factors considered in this study and other sociocultural aspects. An alternative direction would be to administer semistructured interviews that assess attachment representations, such as the Friends and Family Interview (Steele et al., 2009) and the Current Relationship Interview (Crowell and Owens, 1998), to investigate the nature of attachment relationships in adult partnerships. This approach would provide insight into the deeper aspects of the relationship between the caregiver and the partner.

## Ethics Statement

This study was approved by the ethical committee of the “University of Milano-Bicocca,” Italy.

## Author Contributions

PDC: data collection. MDF: consulting and data collection. AnS: supervision and statistical analysis. GT: statistical analysis and writing. AlS: leader of the project and writing.

## Conflict of Interest Statement

The authors declare that the research was conducted in the absence of any commercial or financial relationships that could be construed as a potential conflict of interest.
